# End-to-end deep learning framework for printed circuit board manufacturing defect classification

**DOI:** 10.1038/s41598-022-16302-3

**Published:** 2022-07-22

**Authors:** Abhiroop Bhattacharya, Sylvain G. Cloutier

**Affiliations:** Department of Electrical Engineering, École de technologie supérieure, 1100 Notre-Dame Ouest, Montreal, QC H3C 1K3 Canada

**Keywords:** Electrical and electronic engineering, Software

## Abstract

We report a complete deep-learning framework using a single-step object detection model in order to quickly and accurately detect and classify the types of manufacturing defects present on Printed Circuit Board (PCBs). We describe the complete model architecture and compare with the current state-of-the-art using the same PCB defect dataset. These benchmark methods include the Faster Region Based Convolutional Neural Network (FRCNN) with ResNet50, RetinaNet, and You-Only-Look-Once (YOLO) for defect detection and identification. Results show that our method achieves a 98.1% mean average precision(mAP[IoU = 0.5]) on the test samples using low-resolution images. This is 3.2% better than the state-of-the-art using low-resolution images (YOLO V5m) and 1.4% better than the state-of-the-art using high-resolution images (FRCNN-ResNet FPN). While achieving better accuracies, our model also requires roughly 3× fewer model parameters (7.02M) compared with the state-of-the-art FRCNN-ResNet FPN (23.59M) and YOLO V5m (20.08M). In most cases, the major bottleneck of the PCB manufacturing chain is quality control, reliability testing and manual rework of defective PCBs. Based on the initial results, we firmly believe that implementing this model on a PCB manufacturing line could significantly increase the production yield and throughput, while dramatically reducing manufacturing costs.

## Introduction

The Printed Circuit Boards (PCBs) are the foundation supporting most electronic products. They are usually made of fibreglass and composite epoxies with laminated materials^1^. Any fabrication defect at the PCB level can lead to fatal flaws at the product level. Thus, PCBs must be manufactured with the highest degree of precision to ensure optimal operation and product reliability. With the growing worldwide demand for electronic products, it is essential to detect fabrication defects both efficiently and accurately. As part of the *Industry 4.0* revolution, new data- and machine learning-driven technologies can be implemented to improve product and process quality^2^. The Zero Defect Manufacturing (ZDM) paradigm also aims to improve the manufacturing sustainability by leveraging data-driven methods to ensure no defective products pass through the production process^3^. The approach combines detection, repair, prediction and prevention^4^. While traditional quality improvement (QI) methods focus on the detection-repair, manufacturing industries now migrate towards a prediction-prevention paradigm using data-driven methods to predict manufacturing defects^5^. The PCB industry invests massively to train and maintain a large workforce dedicated to quality inspection using traditional inspection tools^6^. This process often leads to an unwanted latency in the manufacturing process. Moreover, physically inspecting parts is expensive and arduous. Thus, most manufacturing companies rely on batch inspection. However, batch inspection does not enable the manufacturers to comply with the ZDM principle of zero defects at the end of the manufacturing process. With the growing importance of product customization, there is an increase in defect rates due to smaller production batch sizes^7^. In Virtual Metrology(VM), a sub-field of ZDM, data-driven methods help estimate and predict the quality of a product^8^. These methods leverage low-cost quality metrics to derive more complex metrics to achieve a significant improvement in cost efficiency^8^. Emerging machine learning-based computer vision techniques have helped researchers apply Virtual Metrology to quality inspection^9^

### PCB manufacturing defect types

Different types of defects in the copper pattern can afflict the PCBs. They can be *fatal defects*, immediately rendering the device non-functional. They can also be *potential defects*, hindering the performance of the device and reducing its operating lifetime^10^. During the etching and plating processes, anomalies may result in excess copper or missing copper. Also, an incomplete process can result in the unwanted deposition of conductive materials and form defects like shorts or spurs. On the other hand, excessive processing can lead to missing holes, open circuits and mouse bites. Faulty tooling can also produce missing holes. Incorrect timing can lead to mechanical mis-registrations, dirt contamination, or air bubbles from the electrolysis present in bare PCB boards. The literature gives an extensive summary of the most common PCB manufacturing defects and their origins^11^.

### Inspection techniques

The PCB defect detection techniques can be broadly divided into *contact* and *non-contact* methods^11^. Contact methods usually rely on *flying probes* to detect defects causing electrical shortages and open circuits. They can be expensive to implement and maintain. They also have certain limitations which may allow defective products to pass^10^. Defects like spurious copper or burs, which can cause a slower degradation and failure of the board are often missed by electrical contact methods^10^.

The non-contact inspection methods uses several techniques such as X-ray imaging, Scanned Beam Lithography, Ultra-sonic imaging, thermal imaging and Automatic Optical Inspection (AOI)^10^. As the circuits become denser and more complex, the detection of manufacturing defects also becomes increasingly challenging and costly. This prevents the manufacturing process from complying with ZDM standards^4^.

In this work, we seek to improve AOI systems for defect detection in bare-board PCBs using emerging deep-learning techniques. AOI systems can detect defects on bare boards, missing components, soldering and padding defects. They can also detect potential defects such as burs, spurious copper and mouse-bites, which may be missed by contact methods. Moreover, AOI systems avoid mechanical damage and can easily scale with the increase in production capacity.

This paper presents a complete framework to improve PCB defect identification using AOI tools. The main contributions of this paper are as follows:

1. Proposing an improved object detection model for PCB defect detection.
2. Benchmarking its performances against several state-of-the-art object detection models.
3. Increasing the overall accuracy (mAP[IoU = 0.5]) by 3.2%, reaching up-to a 5.6% improvement for the spur-defect class.
4. All this while using only 7 million (7M) (35%) of the more-than 20M parameters used by the current state-of-the-art methods (YOLO V5m for low-resolution images and FRCNN-ResNet FPN for high-resolution images).

In time, we firmly believe such real-time machine learning-assisted monitoring will help rapidly identify and locate manufacturing defects, accurately pinpoint their origin, and provide timely adjustments to the manufacturing processes.

The paper is organized as follows: in “Related work”, we survey the literature with particular attention to the implementation of deep learning for defect detection. In “Overview of objection detection models” the relevant deep learning models are explained. This section also introduces the reader to our proposed model architecture and how it differs from the state-of-the-art. In “Experimentation”, we describe the dataset and the testing methodology. In “Results”, we present the experimental results and ablation studies. The “Discussion” section compares the results using our model and the state-of-the-art, while the “Conclusion” summarizes the key findings and introduces future research directions.

## Related work

Here, we present a brief, yet representative, overview of the field. Zero Defect Manufacturing is a key part of *Industry 4.0*. The zero-defect concept was introduced in 1965 as a quality and reliability program implemented by the US Army^12^. Researchers have explored different techniques to make manufacturing processes compliant with ZDM. In this work, we primarily focus on the application of deep learning for defect detection. Two years ago, researchers implemented an Extended Deep Belief Network (EDBN)-based fault classifier for chemical processes using a combination of raw data and hidden features^13^. However, such an architecture is complex and requires a longer time to process the data. Others proposed a Stacked Quality-Driven Autoencoder (SQAE), which captures quality-relevant features and neglects the irrelevant ones for soft-sensing applications^14^. Transfer convolutional neural networks (CNNs) combine online CNNs and smaller offline shallow CNN networks^15^. This approach shows that pre-training the shallow networks and transferring the knowledge to the online network can significantly improve the accuracy of the models^15^. However, such transfer learning methods tend to introduce unwanted biases in the models, which prevents generalizing across different samples^16^. A central assumption with deep learning-based methods is that the test data and the training data are taken from the same distribution^17^. Concretely, this assumes no change in environmental conditions. As such, deep Transfer Network can achieve better domain adaptation^18^. Indeed, such CNN-based networks were previously used for crack detection on surfaces^19^.

In 2018, Vafeidas et al. performed a comparative analysis of the performance of classical machine learning algorithms to detect faulty component placement on PCB boards^20^. A combination of computer vision algorithms was used to extract the features. At the time, Support Vector Machines achieved the highest classification accuracy^20^. In 2020, an architecture based on 3D convolutional neural networks (3DCNN) was used to simulate the changes in shape and volume of glue drops deposited on Liquid Cystal Polymer substrates before the attachment of integrated circuits^21^.

Virtual Metrology exploits available information from sensors or visual inputs to assess parameters which are difficult or expensive to measure^8,9^. Based on the same paradigm, Autoencoders were trained on defect-free semiconductor chips and, then used for anomaly detection^22^. A similar approach was also used for wafer fault monitoring^23^. The authors showed that the model is able to extract noise tolerant features. However, this process also detects any anomalous sample as a defected sample.

In recent years, researchers used multiple methods to streamline and automate the detection of defects in bare PCBs. Wavelet-based algorithms were first implemented for defect detection^24^. The major limitations with these methods is their poor generalization^25^. Classical machine learning algorithms such as support vector machines and decision trees have been implemented to classify defect types^26,27^. These methods require extensive feature engineering to process the raw data and identify meaningful features for the model. In turn, this leads to unintentional expert-induced bias in the results^16^. This approach requires additional pre-processing steps, increasing the processing time and reducing the scalability. Our proposed deep-learning model seeks to extract the features directly from the images themselves. Indeed, several researchers have used a combination of computer vision methods and deep learning for detecting and classifying defects^28–31^. This also creates pre-processing pipelines, leading to delays and scalability issues. Last year, DETR models removed the need for many hand-designed components for PCB defect detection^32^. Moreover, the use of transfer learning is a popular technique for applying complex deep learning models to small datasets. Recently, a few groups have implemented transfer learning for defect detection^33–35^. However, these models tend to incorporate biases from the pre-training^16^. This year, Generative Adversarial Networks (GANs) were first implemented to improve the quality of data, which in-turn improves the accuracy of the models^36^.

Object detection models can simultaneously perform multiple tasks on a single image, including: (i) Multiple object detection, (ii) Classifying the defects and (iii) Localization^37^. This makes them the ideal deep learning models for application in fault diagnostics. Several research teams are currently exploring object detection models to detect small objects^38,39^. Two-stage object detection models such as FRCNN combine two networks and, thus, have a large number of parameters and require higher image-processing delays. This makes it very difficult to apply them in a high-speed manufacturing line. Single-stage object detectors are faster and thus, more suitable candidates for near real-time deployment. Only last year, researchers started using one-stage object detection models for localization and classification of defects^40,41^. Also, optical inspection requires high resolution images and equipments^42^. This makes it difficult for small scale productions to adopt such technologies. To the best of our knowledge, this is the first extensive study to examine and improve the performances of such models on low-resolution images.

## Overview of objection detection models

This section gives a brief overview of the various models used in the paper. The literature gives a comprehensive overview of the various deep learning methods used for object detection^37^. In this work, we will focus on state-of-art You-Only-Look-Once(YOLO), RetinaNet and Faster R-CNN models for comparison.

### You-only-look-once(YOLO)

You-Only-Look-Once(YOLO)^43^ is a single-stage object detection model. It contains three main components, namely the *backbone*, the *neck* and the *head*. The backbone is a convolutional neural network that takes images of different sizes as input and forms the overall features of the images. The neck represents a series of network layers that can fuse the features to enrich the information. The processed features are fed to the prediction layer, where the classifier obtains the class of the objects and generates the final coordinates of the bounding box.

**Figure 1:**
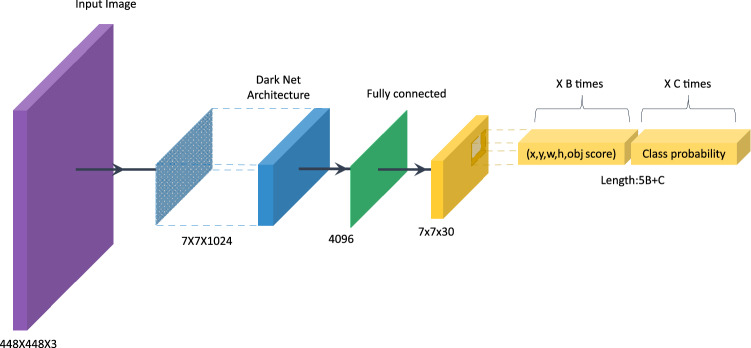
Structure of the YOLO network.

The network divides the image into grid regions and predicts rectangular bounding boxes in each region. The base model for YOLO is similar to GoogLeNet^44^ with the inception module replaced by 1 × 1 and 3 × 3 convolutional layers. The final prediction is produced by two fully connected layers over the whole convolutional feature map. The block diagram in Fig. 1 captures the network structure of YOLO.

The loss function for the network consists of two parts, the *localization loss* for the prediction of the bounding box offsets and the *classification loss* for conditional class probabilities. The losses are computed as the sum of the squared errors. Most of the bounding boxes have no instance of the object, thus it is important to down-weight the loss from the background boxes. Two weight parameters are used to balance between bounding box coordinates and confidence score prediction for boxes without objects.

YOLO v5^45^ is built on a similar structure as YOLO. As such, it runs detection directly over dense location sites. The backbone first extracts the dominant features from the input images. In YOLO v5, the Cross Stage Partial(CSP) Network is used for the backbone. The neck is then used to generate feature pyramid filters. They help the model recognize the same object at different scales and sizes. YOLO v5 uses PANet as the neck^46^. Finally, the head performs the detection part. It applies anchor boxes on the extracted features and produces output vectors with class probabilities, object scores and bounding boxes. YOLO v5 uses Leaky-ReLU for the hidden layers and Sigmoid activation for the output layer. For large models, the stochastic gradient(SGD) optimizer is preferred with a Binary Cross Entropy (BCE) loss^47^. Earlier this year, researchers have applied YOLO-based object detection methods for fault diagnostic^48,49^.

### Faster region based convolutional neural network (FRCNN)

Faster R-CNNs(FRCNNs) combine two networks^50^. First, a Region Proposal Network(RPN) generates region proposals. In turn, a detector network relies on these proposals for object detection. The Faster R-CNN is a significant improvement over it’s predecessor the Fast R-CNN model^51^, as it uses the RPN instead of a selective search method to generate the region proposals. The RPN ranks the region box anchors and proposes the most likely to contain the objects. These anchors play a vital role in the Faster R-CNN models. Typical FRCNNs use nine anchors at each position of an image. The RPN outputs a set of proposals to be further examined by a classifier and regressor to check the object occurrences. Thus, the RPN predicts the probability of an anchor being a meaningful object or being part of the background and then refines the anchor. It then labels as *foreground* the anchors with the greater overlap with the ground-truth boxes. In contrast, the anchors with low overlaps are labelled as *background*. The regressor calculates the L1 loss using the position of the bounding box and positive anchors. The default configuration uses the center position, height and width as the input. However, we observed that using the top left and bottom right coordinates gives a marginally better result. After the RPN, the model proposes regions with different sizes. A Region of Interest(ROI) pooling layer then splits the input feature map into a fixed number of equally sized regions and then applies max pooling on each region to ensure the same region sizes irrespective of the input. We have used ResNet 50 with Feature pyramid networks as the background. The SGD optimizer with a decaying learning rate gives the best results.

### RetinaNet

RetinaNet^52^ is a composite network using a backbone, classification and regression subnet. The typical backbone uses a ResNet with Feature Pyramid Network (FPN)^53^, using two laterally-interconnected pathways. The *bottom-up pathway* uses the output of the final feature map from a set of consecutive convolutional layers. The *top-down pathway* uses nearest neighbour up-sampling to expand the last feature map to the same size as the preceding penultimate layer. These layers are merged by element-wise addition. It then iterates until feature maps from the bottom-up pathway find a corresponding feature map through the lateral connections. This process renders the model scale-invariant. The classification subnet uses a convolutional network (CNN) attached to each FPN. It typically uses four 3 × 3 convolution layers with 256 filters, followed by a ReLU activation. Then, another 3 × 3 convolution layer is followed by sigmoid activation. The classification loss used is a variant of the focal loss^52^. The regression subnet is attached to the FPN’s feature maps in parallel to the classification subnet, akin to a classification network architecture. The RetinaNet typically picks the 1k anchor boxes with the highest confidence score from each FPN level. To prevent redundancy, non-maximum-suppression (NMS) can be applied independently to each class, then choosing the anchor box with the highest confidence score and removing overlapping anchor boxes using Intersection-over-Union (IoU) greater than 0.5^54^. Finally, the regressor performs an offset prediction to refine the anchor selection and return a bounding box prediction.

### Proposed network structure

**Figure 2:**
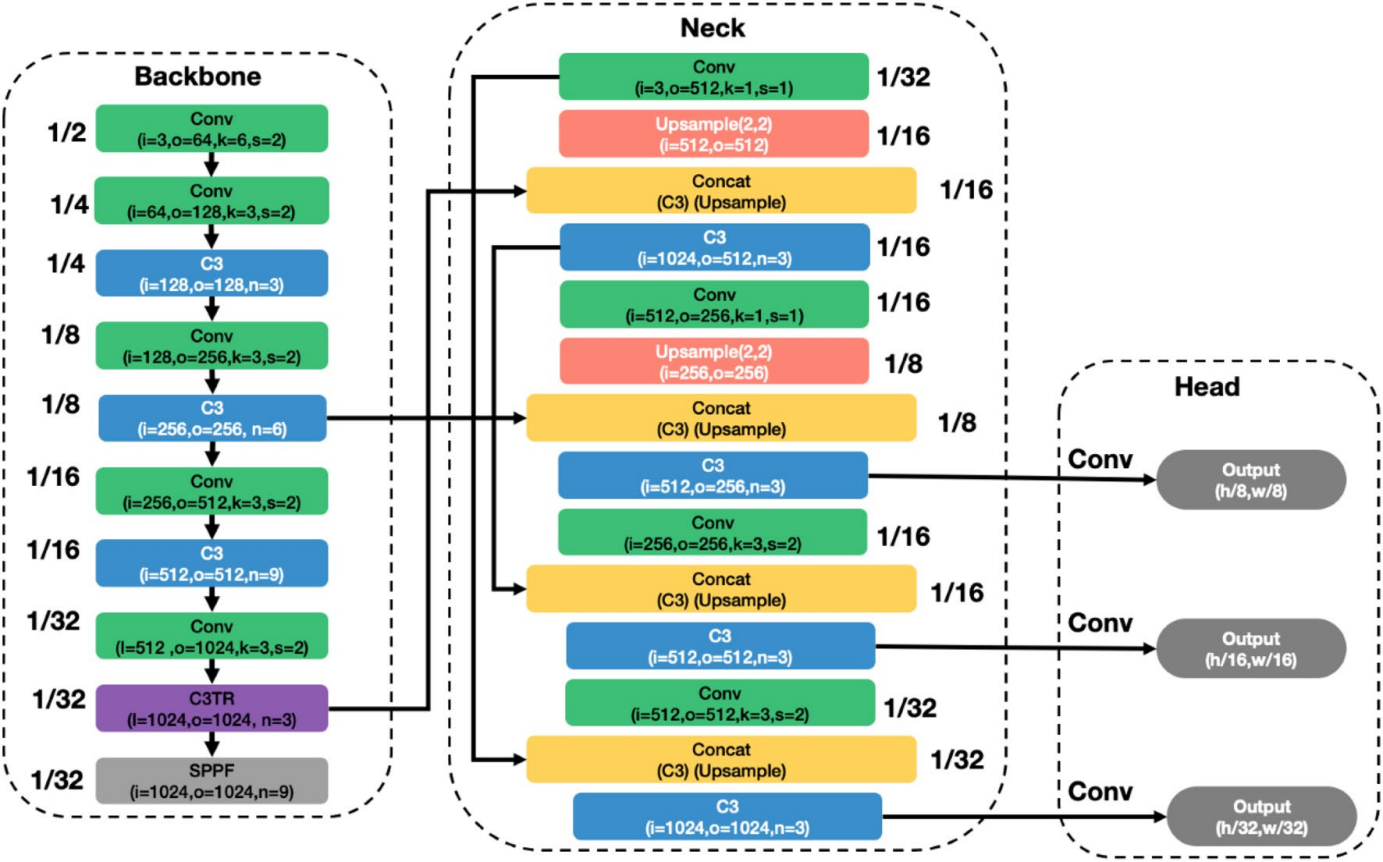
Structure of the proposed model. The structure combines the merits of both transformer and convolutional networks As such, it can exploit global dependencies and locality informations.

The proposed model is a variation of YOLO v5. Our proposed network, is a combination of CNNs and Transformers. Figure 2 shows the model structure, which includes the three main blocks, namely—the backbone, the neck and the head. The transformer module is included at the junction of the neck and the backbone. It provides multi-level features with global information for detection. This enhances the field of reception of the convolutional network. The CNN network extracts the underlying geometrical features of the images. These feature maps usually constitute the key points, lines and some basic geometrical patterns^55^. Both global dependence and locality modelling are important for a better representation of the image^56^. Unlike standard transformer networks used for data sequences, our model directly processes feature maps generated by the convolutional network. As such, our model can benefit from the merits of both CNN and transformers to model long dependencies and, to learn scale combined with shift-invariant locality representations^48,57^.

We present a comparison between the different models analyzed in this paper in the supplementary information section.

### Data augmentation

Data augmentation is an umbrella of techniques that can be used to generate additional training samples by slightly modifying the existing training data or creating synthetic data. It prevents over-fitting and helps the model generalise^58^. Shorten provide a comprehensive survey of different data augmentation techniques used for computer vision^59^. YOLOv5 model uses a combination of Mosaic, Mixup, HSV and classical methods for data augmentation. Classical methods primarily involve rotation, re-scaling, vertical and horizontal flipping, translation, adding noise, cropping and zooming.

The *mosaic* data augmentation technique combines four training images into one single image. This technique was introduced in YOLOv4^60^. This enables the model to learn to identify objects at different scales. The *CutMix* technique combines images by cutting parts and pasting them onto the augmented images. This improves the robustness of the model by changing a part of the input image^61^. On similar lines, *Image Occlusion* replaces regions of the images with random values. This behaves like a regularization technique. Another common method is to increase the Saturation (S) & Value (V) components of the HSV color space.

## Experimentation

We have used a Tesla T4 graphics processing unit (GPU) to run our experiments. The framework was implemented using the PyTorch library version^62^ and the ultralytics YOLOv5^45^ implementation.

**Figure 3:**
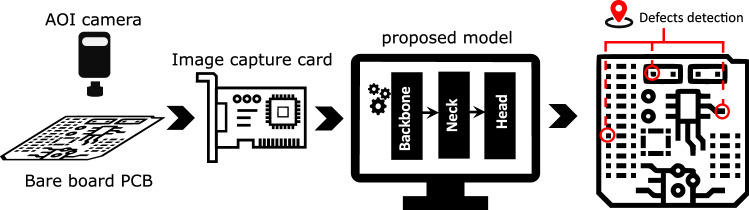
Overall architecture of the model.

The overall architecture involves an AOI camera, an image capture device for storing the images and a processing unit with our proposed model to accurately detect, classify and localise multiple defects in the bare board PCB in real time. Figure 3 presents a high level schematic of the overall architecture.

### HRIPCB dataset

**Figure 4:**
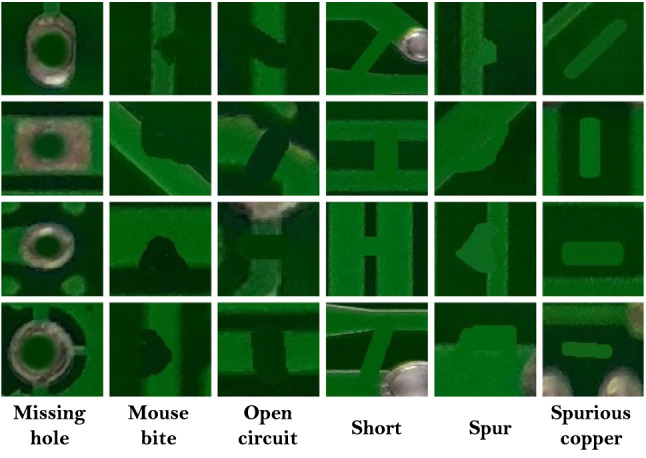
Annotated extracted defect contours (EDCs)^29^.

For this project, we use the public HRIPCB dataset to train, test and validate the object detection model^29^. The dataset contains 1386 labeled images with six different families of manufacturing defects (missing hole, mouse bite, open circuit, short, spur, spurious copper). It is based on 10 different PCB board images augmented using six different defect types^29^. The dataset provides “XML” files with the labels of defects and types. Due to the requirements of the experimental model, the files are converted to “TXT”. Figure 4 shows the different defect types in the dataset.

### Evaluation metrics

In the object detection task, the model will output the possible defect positions in terms of the prediction boxes. To determine whether the prediction box and the ground truth are the same, the metric Intersection over Union(IoU) is used in literature. The definition of IoU is given in Eq. (1):

1 $$IoU = \frac{Area\,of\,overlap}{Area\,of\,union}$$

Most researchers set the threshold of IoU to 0.5. This means that when the overlap ratio reaches 0.5, the prediction box is correct. In addition, average Precision and Recall are used to evaluate the results of image classification. Precision is defined in equation 2 and Recall is defined in equation 3.

2 $$Precision = \frac{True\,Positives}{True\,Positives+False\,Positives}$$

3 $$Recall = \frac{True\,Positives}{True\, Positives+False\,Negatives}$$

The Precision value indicates the model’s *positive predictive value* (or the ability to avoid false positives). Meanwhile, the Recall value indicates the model’s true positive rate or *sensitivity* (or the ability to avoid false negatives). Balancing the Precision and Recall in the context of a specific application is one of the main challenges facing any machine-learning developer.

### Methodology

We first divide the dataset into three training (70%), validation (20%) and test (10%) sets. We used bi-cubic downsampling^63^ to generate the low-resolution images. The high resolution images were also generated using the same method. Bi-cubic sampling does not require any additional learnable parameters. Thus, it does not increase the complexity of the system. The stochastic gradient descent (SGD) optimizer was used for most of the experiments. Additional experiments were performed using the Adam and AdamW optimizers. However, we found that SGD consistently produced the best results. The same behaviour has been previously reported in the literature^47^. The hyperparameters were fine-tuned for each model and the detailed list of hyperparameters associated with each model is provided in the supplementary information. For fine-tuning the hyperparameters of our proposed model, we have used a genetic algorithm-based approach^64^.

The YOLO v5 algorithm uses a combination of different methods for data augmentation. Our proposed model uses a combination of image HSV augmentation, translation, rotation, mixup and mosaic. The detailed description of each augmentation method is provided in the section “Data augmentation”. The Faster R-CNN model is trained using a ResNet50 and a MobileNetv2^65^ backbone for extracting the features. The RetinaNet model is trained using a ResNet50 feature extractor. We experimented with ResNet50 and GhostNet backbone models for YOLOv5s. However, we find that Cross Stage Partial Networks (CSP) Backbone gives the optimal results. For the Neck, we ran experiments with BIFPN, FPN and PANet models. The outcome of the experiments are presented in the section “Results”.

**Figure 5:**
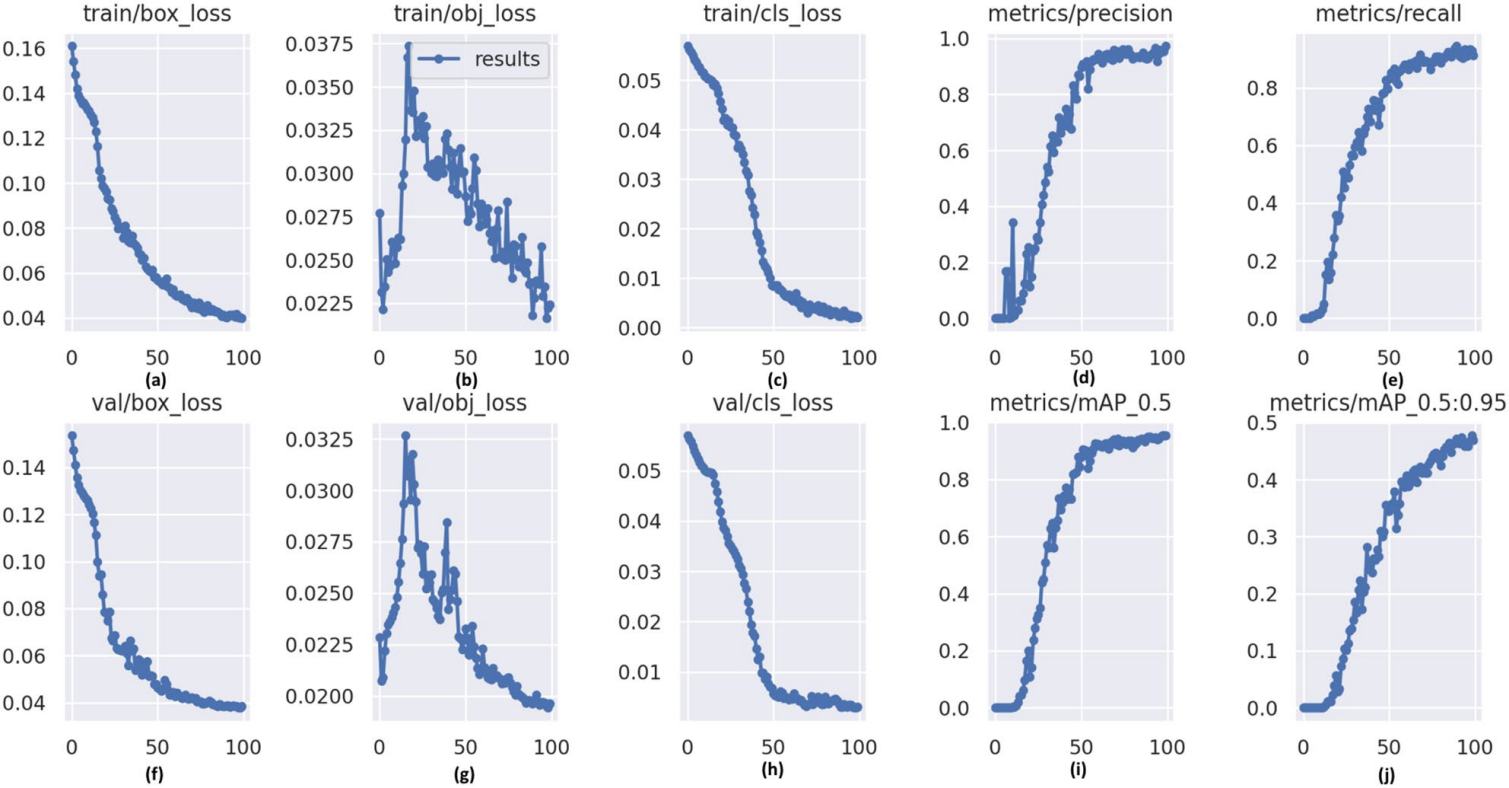
The training and validation curves for our model using low-resolution images. Plots (**a**–**c**) show the box loss, object loss and classification loss for the training sample. Figures (**f**–**h**) capture the same metrics for the validation dataset. The figures (**d**) and (**e**) show the precision and recall of the model. The figures (**i**) and (**j**) capture the mAP at IoU = 0.5 and IoU = 0.5:0.95 respectively. For each figure, the x-axis represents the number of epochs.

The key improvement in our model is the addition of the transformer model. The network structure is described in the section “Proposed network structure”. We also ran additional experiments using different activation functions. We found that the Swish activation function performs better than Mish, ReLU and Leaky-ReLU. Figure 5 shows the training and validation curves for our model. It demonstrates that our model is able to converge within 100 epochs for low-resolution images.

## Results

### Experimental results

Object detection performance is evaluated with the mean Average Precision (mAP) between ground truth and predicted bounding box (IoU). To validate the performance of our proposed network, multiple experiments are performed in this study. To benchmark the performance of different object detection models against our proposed model, we coded the models as per the respective references and then ran the experiments on the HRIPCB dataset.

Figure 5 shows that the performance of the model is the same across training and validation, which suggests that the model does not over-fit the data. We observe that the box loss and the classification loss decrease sharply for the first 50 epochs before saturation. Also, we observe that the model has a high precision and a high recall, which suggests that the model has low false negatives.

Experiment results reported in the Table 1 are the average of multiple experiments.We performed two sets of experiments to compare model performances: one using high-resolution images and the other using low-resolution images. We use bi-cubic sampling^63^ to generate the low resolution images. The results in Table-1 show that our method can achieve an overall Mean Average Precision (mAP[*IoU* = 0.5]) of 98.1% on low-resolution images. We observe that the two-stage object detection FRCNN model with ResNet back bone is able to achieve a high performance. However, the model has a large number of parameters and is significantly slower than single-stage object detectors^38^. Amongst the single-stage models, we observe that the YOLOv5 models outperform RetinaNet and YOLOv3. As expected, we observe that the medium YOLOv5m model performs better than the smaller YOLOv5s model. We also observe our model outperforms the state-of-art YOLOv5m medium model by 3.2% in the overall mAP[*IoU* = 0.5]. The mAP for IoU = 0.5:0.95 is also higher for our model compared to all other models. This means that the model is able to accurately detect defects for different IoU thresholds, from 0.5 to 0.95 with a step of 0.05.

**Table 1:** Mean average precision (mAP) for PCB defect classification across models.

Method	Resolution	Epochs	Parameters (M)	mAP [IoU = 0.5] (%)	mAP [IoU = 0.5:0.95] (%)
FRCNN-ResNet FPN	High	10	23.59	96.7	51.5
FRCNN MobileNetv2	High	10	2.26	41.8	12.4
RetinaNet	High	10	36.4	93.1	44.7
YOLO V3	Low	100	61.5	92.5	44.2
YOLO V5m	Low	200	20.8	94.9	48.5
YOLO V5s	Low	100	7.02	94.5	48.1
Our model BIFPN	Low	100	7.06	98.3	54.1
Our model PANet	High	100	7.02	96.8	49.5
**Our model PANet**	**Low**	**100**	**7.02**	**98.1**	**53.8**

The values in bold represent the results of the proposed model.

To be useful, a detection system should be able to generalize and accurately detect all types of defects. Indeed, we observe that other YOLO-based models are ill-suited for the spur, mousebite and open circuit defects detection. The smaller YOLOv5s model achieves only 88.5% mAP for spur and 97.4% for open circuit. The state-of-art YOLO v5m medium model achieves only 91.3% mAP for the spur defect detection and 91.6% mAP for open circuit. Our model outperforms the YOLO v5m model to achieve 96.9% mAP on spur defects and 99.5% on open circuit defects. While achieving significantly better performances, our model uses roughly 3× fewer parameters (7.02M) compared with the state-of-the-art FRCNN-ResNet FPN (23.59M) and YOLO V5m (20.08M).

Table 2 presents a comparative overview of the results achieved using the state-of-the-art YOLO 5-based models and our model.

**Table 2:** Mean average precision (mAP) achieved with IoU = 0.50 for PCB manufacturing defect classification across defect types.

Method	Missing hole (%)	Mouse bite (%)	Open circuit (%)	Short (%)	Spur (%)	Spurious copper (%)
YOLO V3	99.1	91.2	92.1	97.3	81.4	93.8
YOLO V5m	99.5	92.3	91.6	97.4	91.3	97.1
YOLO V5s	98.8	95.1	97.4	96.3	88.5	90.5
Our model BIFPN	98.9	97.5	99.5	99.5	98.3	95.9
Our model PANet	**98.4**	**97.2**	**99.5**	**99.5**	**96.9**	**96.8**

The values in bold represent the results of the proposed model.

**Figure 6:**
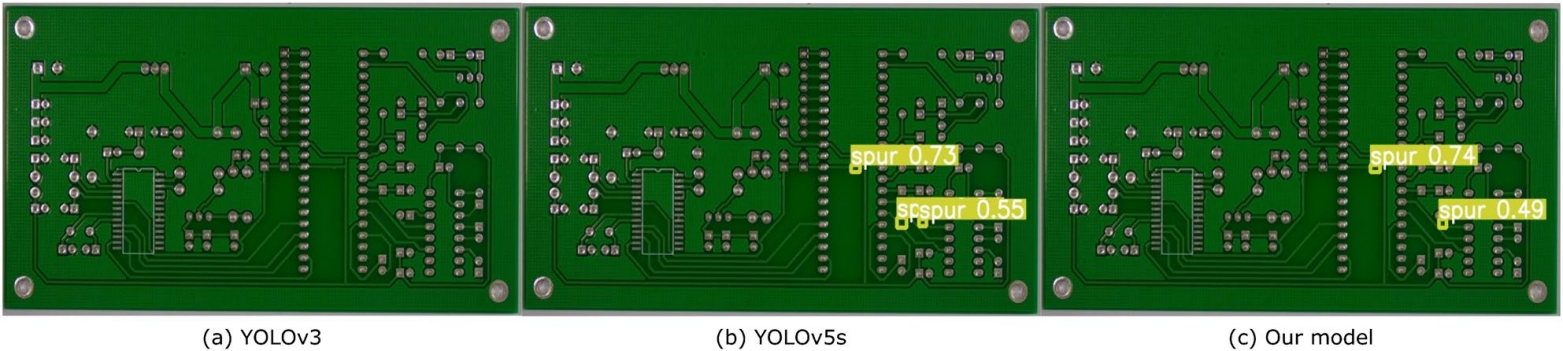
We detected the defects on the bare board PCB using three different models. The studied PCB has 2 spur defects. We observe that YOLOv3 fails to identify the presence of both the spur defects while, YOLOv5s predicts an additional incorrect defect. Our proposed model is able to identify the two correct spurs.

In the example shown in Fig. 6, we observe that the bare board PCB has 2 spur defects. YOLOv3 fails to identify the presence of both the spur defects while the YOLOv5s model predicts an additional false positive. Our proposed model is able to correctly identify both the spurs.

Now, the next section will present a complete ablation study for the different components of the model.

### Ablation studies

#### Data augmentation

The YOLOv5 model uses a combination of augmentation methods for improving its generalization. These methods are discussed in the section “Data augmentation”. We observe that removing the mosaic method does not impact the overall precision. However, it slightly reduces the precision for the spurs and spurious copper defects. If we remove the HSV augmentation, we observe a significant decrease (7%) in the detection precision for spur defects. Furthermore, removing the scale augmentation only has a marginal effect on the overall precision. However, removing all data augmentation drastically reduces the overall mAP to 84.7% and the average precision for detecting spur defects to 73.6%. Thus, we see that data augmentation has a significant impact on the performance of the model.

#### Neck

**Figure 7:**
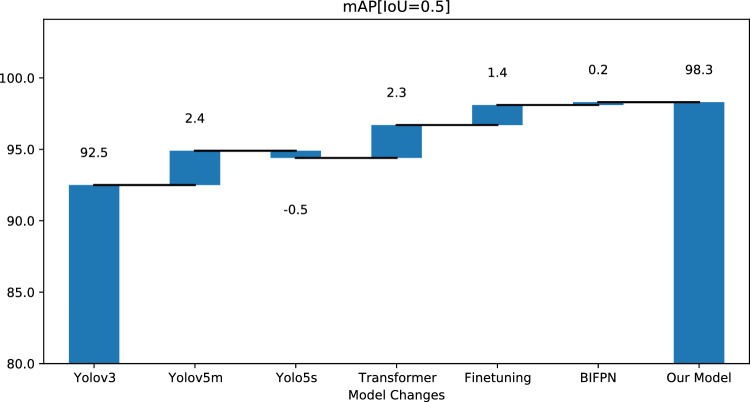
Mean average precision (mAP) waterfall chart showing that we find a significant improvement when we change in the model architecture. The maximum improvement can be attributed to the transformer module. Our model in the chart refers to the proposed model with BIFPN neck.

We have removed the Path Aggregation module and used a BiFPN model for comparison. We observe that the BiFPN model yields similar results. The overall accuracy increases by 0.2% and we observe an increase of 1.4% for the spur defects. Thus, we observe that BiFPN marginally improves the performance of the system at the cost of a slight increase in compute parameters. The waterfall chart in Fig. 7 captures the effect of each model change on the average precision.

#### Activation functions

**Figure 8:**
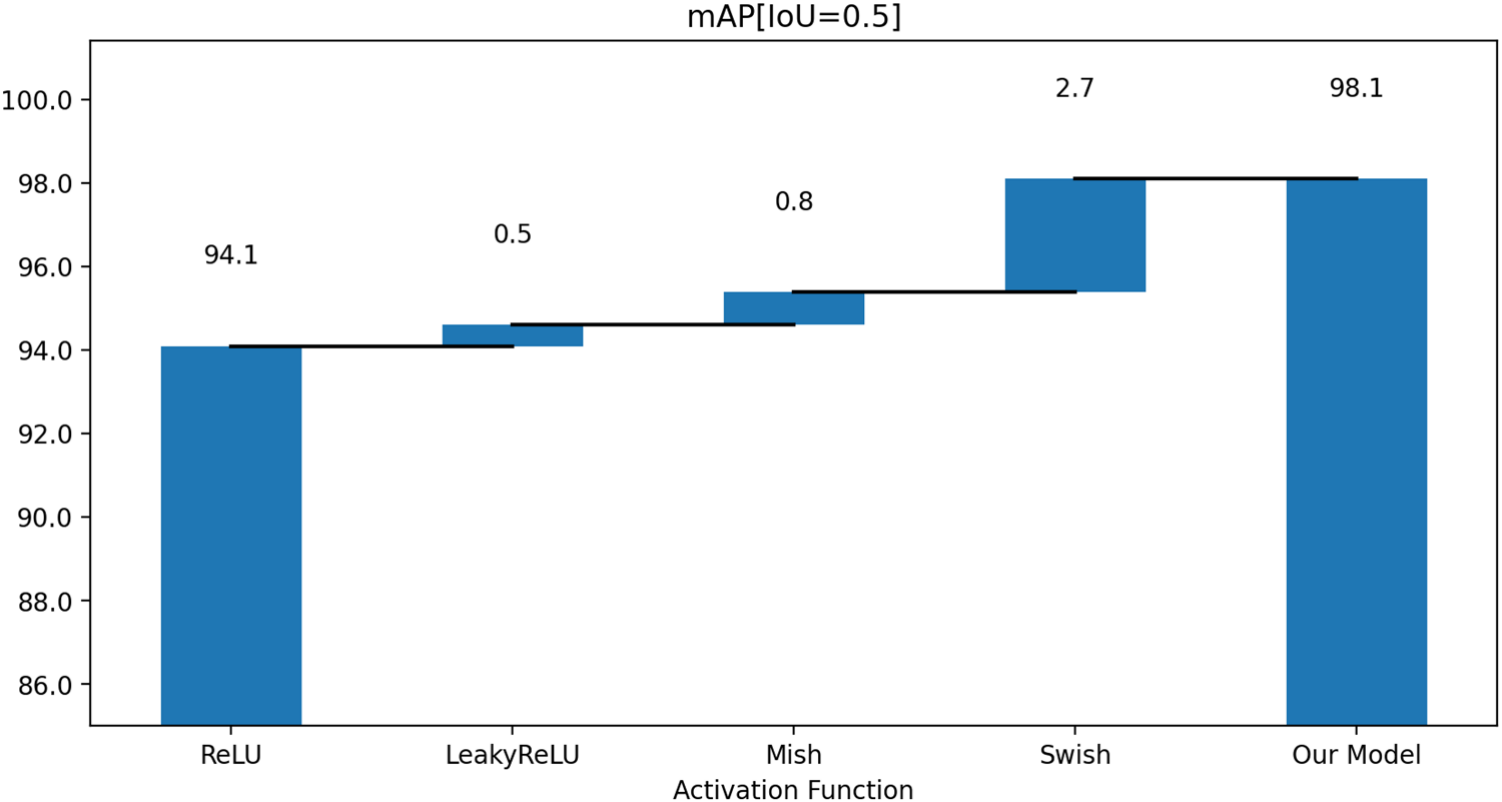
Mean average precision (mAP) waterfall chart showing the effect of changing the activation functions on the model. We find a significant improvement when we use the Mish activation function. The Swish activation function yields the best improvement in our model.

We have also compared the performances of the model using different activation functions. We have changed the activation function for the convolutional layer. We observe that using a ReLU activation function reduces the accuracy, as the ReLU function cannot recover after getting stuck in a negative region. The Leaky ReLU performs slightly better. However, we see that the Swish activation function outperforms the other activation functions. The waterfall chart presented in Fig. 8 captures the effect of using the different activation functions on the model’s mean average precision (mAP).

#### Regression loss functions

**Figure 9:**
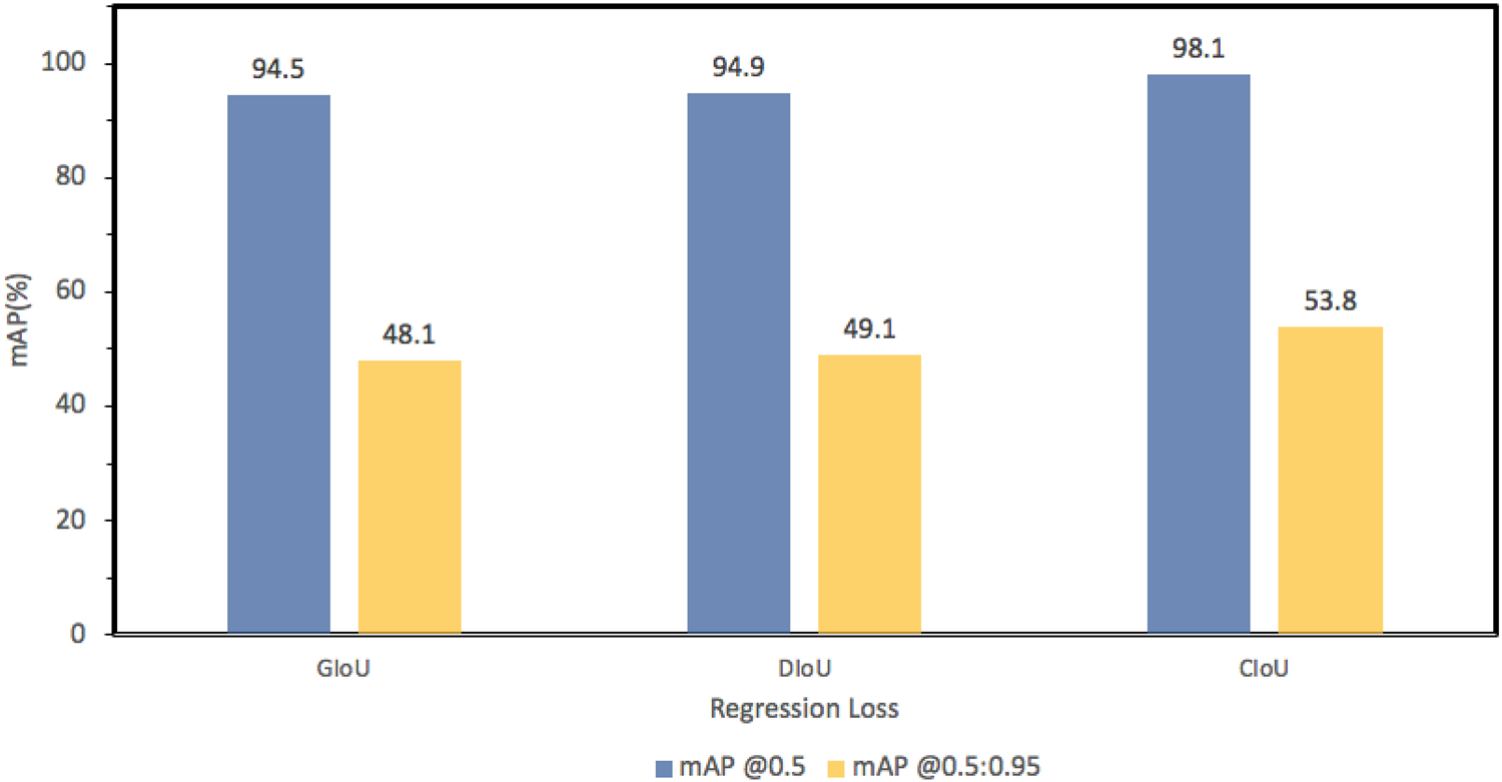
Regression loss comparison (RLC) showing the model’s precision (mAP) using the different regression loss functions. The CIoU loss clearly outperforms the others.

The regression loss function is a key factor in the training and optimization process of object detection. The most widely used regression loss function is the Smooth Ln-norm^66^. There are some limitations associated with the Ln-norm loss. For example, they cannot combine the parameters of the bounding box^67^. The IoU loss offers significant improvement over the Ln norm loss^68^. It is based on the cross-union ratio between the bounding box and ground truth. Thus, it is scale invariant. However, for certain instances when the IoU score of the ground truth and the bounding box is zero, the loss suffers from a problem of vanishing gradient. Silvio et al. proposed Generalized IoU(GIoU) loss to address the limitations in IoU^69^. GIoU uses a penalty term to prevent the IoU loss to keep expanding the size of the predicted box until it overlaps with the target box. However, GIoU can suffer from slow convergence. As such, Zheng et al. show that directly minimizing the normalized distance between the predicted box and the target box helps the algorithm to converge much faster^67^. Moreover, it also considers the vertical and horizontal orientations. This new loss function is referred-to as Distance Intersection over Union(DIoU) loss. However, DIoU is unable to capture the consistency of aspect ratios for bounding boxes. Complete IoU (CIoU)^67^ regression loss incorporates all geometric factors. CIoU works by adding a penalty factor $=\alpha \cdot V$ to DIoU, where *V* represents the aspect ratio consistency. We have compared the effect of those different regression loss functions to find that CIoU significantly outperforms all other loss functions. Figure 9 compares the model’s performance (mAP) using the different loss functions.

In addition to comparing the mainstream loss functions, we also propose a new loss function named AIoU which enables the model to optimise the height and width of the bounding box and the ground truth. We present a detailed analysis of this loss function in the supplementary information.

#### Transformer

Finally, we can run another ablation study by removing the transformer module from the architecture. We observe an overall reduction in accuracy of around 3.4%. However, the deterioration in performance is much more drastic for certain defect types. Compared to the YOLOv5s model, we observe a deterioration of around 9.8% in the mAP for the spur defects detection and a deterioration of 5.4% percent for spurious copper. The waterfall chart in Fig. 7 captures the effect of each model change on the average precision. It confirms that the transformer module is a key component of the framework.

## Discussion

In this section we discuss the results from multiple perspectives. The results show that our model is able to accurately detect, localize and classify multiple occurrence of defects in images of bare board PCBs. The model accurately predicts the location of each defect, classifies the type of defect and provides a probability score.

To understand the potential impact of this work, one must understand how the testing and rework process is performed in most traditional industrial PCB manufacturing lines. A direct-contact (flying probe) test station provides a pass/fail diagnostic on each individual PCB. When it fails, a PCB is sent to the rework station, where a technician will first look for defects under a digital microscope and perform manual rework if possible. If the reworked PCB still fails, it will be sent for a more advanced diagnostic (using tools like X-ray tomography imaging). Some randomly-selected PCB samples with a *pass* diagnostic will also be sent to rework inspection and advanced diagnostic for quality control. This is done to detect those non-fatal *potential defects*, which can impact the long-term device operation and lifetime. With a high-performance and trustworthy model such as the one described in this work, the rework technician could receive a faulty PCB knowing exactly what are the defects and where they can be found on the PCB. Using more advanced analysis methods, one could also potentially pinpoint the origin of those defects in the manufacturing process. As such, a high probability score validated in the field could help manufacturing companies bolster their confidence in the machine learning-enhanced diagnostic tool.

Firstly, we would like to emphasize that the FRCNN with the ResNet backbone can achieve reasonable performances. However, it is a two stage object detector which takes a much longer time to process the images and uses around 23.5M parameters^38^. Thus, we believe it might be difficult to implement this network in high-speed manufacturing lines. While achieving higher precision, the proposed model uses only 7.02M parameters. This is roughly 3× less than the 23.5M parameters used with a two-stage detector or the 20.8M parameters used by the state-of-the-art YOLO5m model.

Secondly, we would like to highlight that the proposed model maintains high detection and identification accuracy with low-resolution images. This allows the technology to be easily adopted across multiple industries as it does not require expensive imaging technology. Moreover, our model yields a 3.2% overall improvement in the mAP[IoU = 0.5] compared to the standard YOLOv5m model for the same duration of training.

Most importantly, the YOLO v5m model achieves only 91.3% mAP for the spur defect detection. Meanwhile, our model achieves 96.9% mAP on spur defects. We believe that this shows that our model is able to accurately predict defects which are difficult to classify. This might be attributed to the ability of our model to exploit both global dependence and shift/scale invariance in extracted features.

The ablation studies show that the combination of data augmentation techniques helps the model generalize and improves the mAP of the model. This prevents the model from over-fitting to the data. The ability to work in high-speed environments with a small datasets is crucial for large scale deployment of the technology. Thus, we prefer the model with PANet, even though it has marginally lower overall performance compared to BiFPN. Furthermore, the model with BiFPN has lower accuracy than PANet for spurious copper, which is a common defect during the etching process. The ablation studies also sheds light on the importance of using the correct activation and loss functions to maximize the performance of the model for accurate defect detection. Table 3 presents a comparative overview of the models.

**Table 3:** Comparative overview of key properties across models.

Properties				
Model	Rapid	Lightweight	Accuracy and diversity	Low resolution
Proposed model	✓	✓	✓	✓
YOLOv5m	✓		✓	
YOLOv3	✓			
FRCNN			✓	

## Conclusion

In this paper, we propose an end-to-end framework to detect manufacturing defects in PCB boards. In an optimal manufacturing process, quality should be integrated into the process. While most fabrication defects stem from the manufacturing process itself, traditional pass/fail methods focus on reworking those defects on the failing PCBs instead of identifying their precise origins in order to rapidly correct the process. Our proposed framework will enable the fabrication units to make *in-operando* adjustments to the manufacturing process and move closer to a zero defect manufacturing paradigm. Our network uses a combination of current techniques in deep learning- transformers, multi-level feature fusion, data augmentation and object detection. The results show that our model is able to successfully detect, classify and localize multiple defects in low resolution bare board PCB images. Our model is lightweight, low- resolution compatible and provides a 3.2% overall improvement in the mAP[IoU = 0.5] compared to the standard YOLOv5m model. Based on the initial results, we believe integrating deep learning-based models into fully-automated optical inspection (AOI) tools is crucial for early detection of PCB manufacturing defects and could potentially lead to important productivity gains coupled with significant cost reductions.

The presented approach has a strong assumption that the test and the training data are sampled from the same distribution. An interesting area of future work could be to investigate the performance of the method for off distribution samples. In the current work, we have only considered single layer bare board PCBs. Another interesting area of development could be extending the analysis for detecting defects in multi-layered PCBs.

## Supplementary Information

Supplementary Information.

## Data Availability

The datasets generated and/or analysed during the current study are available in the *PKU-Market-PCB* repository, http://robotics.pkusz.edu.cn/resources/dataset/.
