# Supplementary material for: End-to-end deep learning framework for printed circuit board manufacturing defect classification
Source: Sci Rep. 2022 Jul 22;12:12559. doi: 10.1038/s41598-022-16302-3 (PMC9307836; doi:10.1038/s41598-022-16302-3)
Supplement: Supplementary file 1 — Supplementary Information. [file 41598_2022_16302_MOESM1_ESM.pdf]

End-to-End Deep Learning Framework for PCB Manufacturing Defect Classification: Supplementary Information

Abhiroop Bhattacharya   Sylvain G. Cloutier*

SUPPLEMENTARY INFORMATION

Improved Bounding box regression loss Function

The regression loss is a key component of the optimisation process of object detection. We propose a new loss function *AIoU* for object detection. The current mainstream loss functions GIoU loss[1] , DIoU loss and CIoU loss[2] have certain limitations. The proposed loss function is described below as per the standard nomenclature in the cited works.

$$AIoULoss = IoU - ((\rho^2(B, B^{gt})/c^2) + \alpha * v + \gamma * ((\rho^2(W, W^{gt})/c_w^2) + (\rho^2(H, H^{gt})/c_h^2))) \tag{1}$$

where,

$$v = \frac{4}{\pi^2}(\log\frac{w^{gt}}{h^{gt}} - \log\frac{w}{h})^2 \tag{2}$$

$$\alpha = \frac{v}{(1 - IoU + v)} \tag{3}$$

$$\gamma = \frac{(w_1 * h_1 - w_2 * h_2)}{c_w * c_h} \tag{4}$$

$$\rho^2(A, B) = (A - B)^2 \tag{5}$$

The proposed loss introduces a new geometric factor which incorporates the euclidean difference between the width and height of the target box and the bounding box. Thus, it allows the algorithm to optimise the difference in the width and height of the boxes. The value of γ is based on the normalized difference in area between the target and bounding box.

As described in the ablation studies, we studied the effect of different regression losses as a part of our ablation studies and observe that the CIoU loss outperforms the other loss functions. We present here a detailed comparison between our proposed loss function and other loss functions.

We have run the experiments using the methodology mentioned in the main document for all the loss functions. We observe that our loss function is able to quickly achieve an higher mean average precision (mAP) for both IoU[0.5] and IoU[0.5:0.95] at a much lower number of epochs. This is crucial for any resource-constrained environments. The figureS1 shows a comparison between the mean average(mAP) precision values.

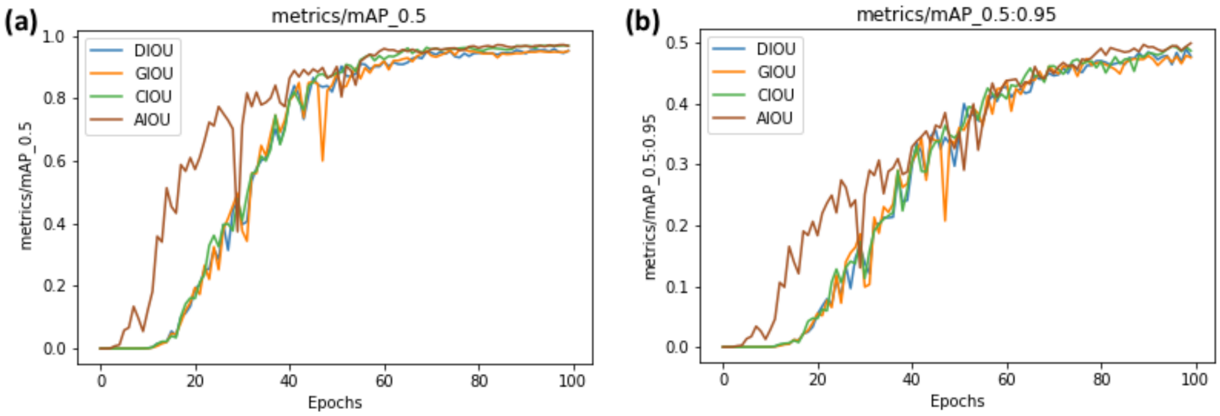

Figure S1. Comparison between the mean average(mAP) precision values using the different loss functions. We observe that our proposed loss(AIoU) achieves a higher precision at a lower number of epochs.(a) Mean Average Precision(mAP)[IoU=0.5] (b)Mean Average Precision(mAP)[IoU=0.5:0.95]

AIoU leads to a much lower box loss and classification loss. However, it leads to an increase in object loss. We observed the same behaviour for the training and validation sample. The figureS2 presents a comparative overview of the losses during training for the different loss functions.

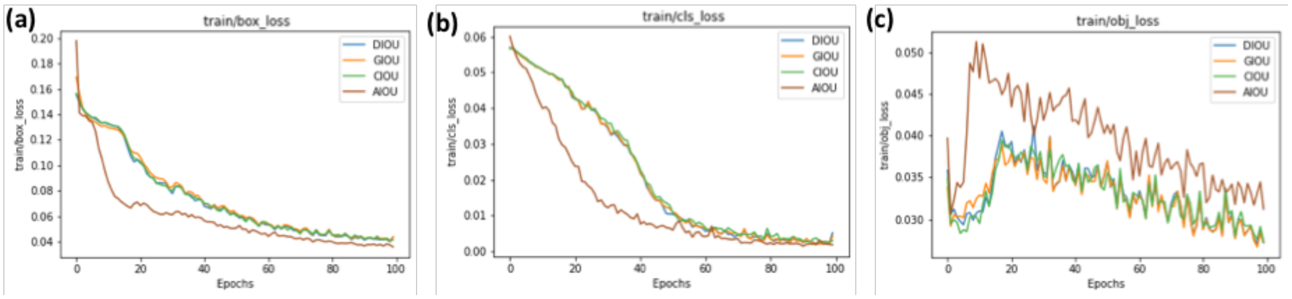

Figure S2. AIoU leads to rapid drop in both the classification loss and Box loss. However, we observe that it increases the value of the object loss. (a)Comparison of Box Loss during training (b)Comparison of Classification Loss during training (c)Comparison of Object Loss during training

We observe that in certain rare instances AIOU fails to identify certain defects which are captured by CIOU. We hypothesize that this is due to the increase in object loss. The figureS3 shows such a rare instance.

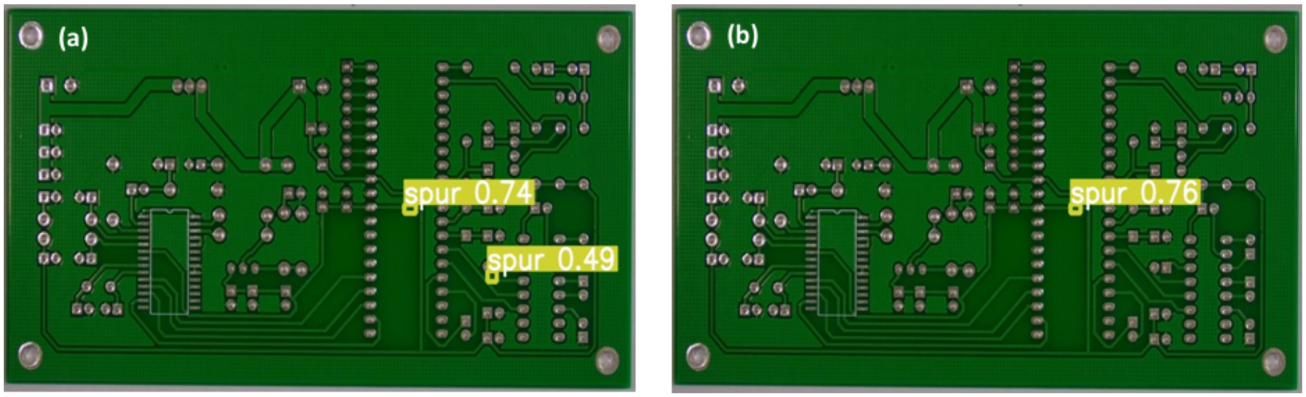

Figure S3. AIoU is unable to detect the second spur. CIoU is able to detect the second spur as well. However, the confidence for the second detection is low. (a)Defect prediction using CIoU loss (b)Defect prediction using AIoU loss.

Transformer Module

Transformers have been widely applied for processing sequential data in the field of Natural Language processing. Transformer models often use transfer learning wherein they are pre-trained on a large model and then fine-tuned[3,4].

Vision transformers have explored the application of transformers to image processing[5]. A central issue is that the model fails to scale to real images as each pixel attends to all the other pixels. To address this problem, the image is flattened and transformed into 2D patches. In addition to the patch embeddings, the model uses position embeddings to incorporate positional information. The block diagramS4 presents a comparison between the models.

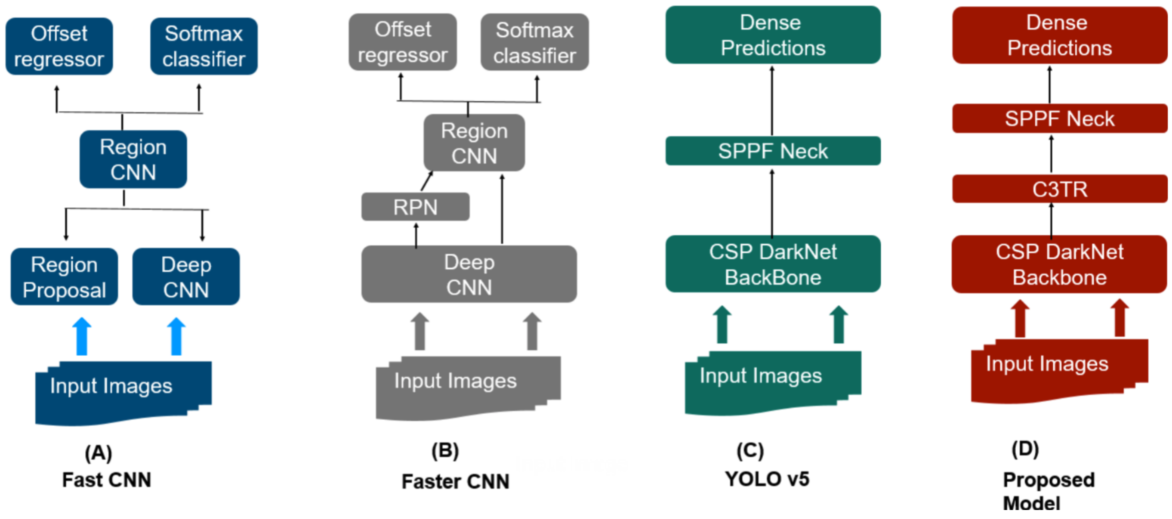

Figure S4. Comparison between the different model architectures at the block-diagram level.

Model Hyperparameters

The following tableS1 outlines the hyperparameters used in our model.

Table S1. Hyperparmeter Values

HyperParameter	Value
Learning Rate- Initial	0.01501
Learning Rate -Final	0.11374
Momentum	0.98
Weight Decay	0.00062
Warmup Epochs	1.6404
Warmup Momentum	0.95
Warmup Bias	0.09243
Box	0.04922
Cls	0.51498
Cls PW	1.073
Obj	1.392
Obj PW	1.1462
IoU Threshold	0.2
Anchor Threshold	4.18357
FL Gamma	0.0
HSV H	0.01266
HSV S	0.50743
HSV V	0.22507
Degrees	0.0
Translate	0.10735
Scale	0.50684
Shear	0.0
Perspective	0.0
Flip UD	0.0
Flip LR	0.5
Mosaic	1.0
Mixup	0.0
Copy Paste	0.0
Anchors	2.41222

References

1. Rezatofighi, H. *et al.* Generalized intersection over union: A metric and a loss for bounding box regression. In *Proceedings of the IEEE/CVF conference on computer vision and pattern recognition*, vol. 1, 658–666, DOI: https://doi.org/10.1109/CVPR.2019.00075 (2019).

2. Zheng, Z. *et al.* Distance-iou loss: Faster and better learning for bounding box regression. In *Proceedings of the AAAI Conference on Artificial Intelligence*, vol. 34, 12993–13000, DOI: 10.1609/aaai.v34i07.6999 (2020).

3. Devlin, J., Chang, M.-W., Lee, K. & Toutanova, K. Bert: Pre-training of deep bidirectional transformers for language understanding. *arXiv preprint arXiv:1810.04805* DOI: https://doi.org/10.48550/arXiv.1810.04805 (2018).

4. Floridi, L. & Chiriatti, M. Gpt-3: Its nature, scope, limits, and consequences. *Minds Mach.* **30**, 681–694, DOI: 10.1007/s11023-020-09548-1 (2020).

5. Dosovitskiy, A. *et al.* An image is worth 16x16 words: Transformers for image recognition at scale. *arXiv preprint arXiv:2010.11929* DOI: https://doi.org/10.48550/arXiv.2010.11929 (2020).
